# A mixture of quebracho and chestnut tannins drives butyrate-producing bacteria populations shift in the gut microbiota of weaned piglets

**DOI:** 10.1371/journal.pone.0250874

**Published:** 2021-04-29

**Authors:** Francesco Miragoli, Vania Patrone, Aldo Prandini, Samantha Sigolo, Matteo Dell’Anno, Luciana Rossi, Mario Barbato, Alice Senizza, Lorenzo Morelli, Maria Luisa Callegari

**Affiliations:** 1 Biotechnological Research Centre, Università Cattolica del Sacro Cuore, Cremona, Italy; 2 Department for Sustainable Food Process (DiSTAS), Università Cattolica del Sacro Cuore, Piacenza, Italy; 3 Department of Animal Science, Food and Nutrition (DIANA), Università Cattolica del Sacro Cuore, Piacenza, Italy; 4 Department of Health, Animal Science and Food Safety, Università degli Studi di Milano, Milano, Italy; INRAE, FRANCE

## Abstract

Weaning is a critical period for piglets, in which unbalanced gut microbiota and/or pathogen colonisation can contribute to diseases that interfere with animal performance. Tannins are natural compounds that could be used as functional ingredients to improve gut health in pig farming thanks to their antibacterial, antioxidant, and antidiarrhoeal properties. In this study, a mixture of quebracho and chestnut tannins (1.25%) was evaluated for its efficacy in reducing the negative weaning effects on piglet growth. Microbiota composition was assessed by Illumina MiSeq 16S rRNA gene sequencing of DNA extracted from stools at the end of the trial. Sequence analysis revealed an increase in the genera *Shuttleworthia*, *Pseudobutyrivibrio*, *Peptococcus*, *Anaerostipes*, and *Solobacterium* in the tannin-supplemented group. Conversely, this dietary intervention reduced the abundance of the genera *Syntrophococcus*, *Atopobium*, *Mitsuokella*, *Sharpea*, and *Prevotella*. The populations of butyrate-producing bacteria were modulated by tannin, and higher butyrate concentrations in stools were detected in the tannin-fed pigs. Co-occurrence analysis revealed that the operational taxonomic units (OTUs) belonging to the families *Veillonellaceae*, *Lachnospiraceae*, and *Coriobacteriaceae* occupied the central part of the network in both the control and the tannin-fed animals. Instead, in the tannin group, the OTUs belonging to the families *Acidaminococcaceae*, *Alcaligenaceae*, and *Spirochaetaceae* characterised its network, whereas Family XIII Incertae Sedis occupied a more central position than in the control group. Conversely, the presence of *Desulfovibrionaceae* characterised the network of the control group, and this family was not present in the network of the tannin group. Moreover, the prediction of metabolic pathways revealed that the gut microbiome of the tannin group possessed an enhanced potential for carbohydrate transport and metabolism, as well as a lower abundance of pathways related to cell wall/membrane/envelope biogenesis and inorganic ion transport. In conclusion, the tested tannins seem to modulate the gut microbiota, favouring groups of butyrate-producing bacteria.

## Introduction

Tannins are natural polyphenolic compounds that are widely distributed in plants, mostly in the wood of several tree species. Because of their heterogeneous composition and chemical properties, tannins are divided into five categories, namely gallotannins, ellagitannins, complex tannins, phlorotannins and condensed tannins [1]. The need to reduce the use of antibiotics as a prophylaxis mean to treat post-weaning diarrhoea prompted researchers to search for alternatives. Tannins could represent a natural alternative to antibiotics, and their biological activities make them suitable for that purpose [2]. Tannins from chestnut (*Castanea vesca*) are frequently used as feed supplements for pigs, whereas quebracho (*Schinopsis lorentzii*) tannins or mixtures of both have been evaluated in other animal species, such as poultry, sheep, rabbits, and cows [3–6]. Tannins from chestnut are defined as hydrolysable, while those from quebracho are categorised as condensed. This classification is based on their sensitivity to hydrolysis, the type of monomer unit or the degree of polymer condensation [7].

Traditionally, tannins were regarded as anti-nutritional factors in monogastric and ruminant farm animals responsible for reduced feed digestibility and worse animal performance [8,9]. Thereafter, some studies have shown that tannins can be used in ruminants and monogastrics, particularly in pigs, without any detrimental effects on animal growth. Chestnut tannins significantly improved feed efficiency and growth performance when supplemented in the diet of weaned piglets [10]. Galassi et al. [11] also reached the same conclusion using chestnut tannins as a dietary supplement in heavy pigs since no anti-nutritional effects were observed. However, animals might not find tannin-containing feed palatable due to its astringent taste. Thus, it is crucial to establish the correct percentage of tannins to add to the diet in order to avoid a reduction in feed intake. Notably, in monogastric animals, as compared with ruminants, the dosage can be reduced and still have positive effects. Apart from these application problems, tannins are recognised to have antimicrobial, antioxidant, and antidiarrhoeal properties [12–16]. These properties can be extremely useful in the weaning of piglets, which represents a critical period in the animal’s life. Indeed, several types of stress can be caused by the removal of piglets from their mother, the sudden change in their diet, and the crowding of the farm environment. All these factors can contribute to the development of diarrhoea and a slowdown in growth. In particular, diarrhoea can be due to an unbalanced gut microbiota caused by the introduction of solid feed [17] or the colonisation of pathogenic agents [18–20]. Indeed, encouraging results have been obtained using chestnut tannins to prevent diarrhoea in piglets infected with an *Escherichia coli* ETEC strain. The feed supplementation reduced the incidence and severity of diarrhoea with no negative effects on animal performance [21]. Chestnut tannins were also used as an alternative to zinc oxide in weaning piglets, resulting in improved animal health and a reduction in diarrhoea by means of multifactorial mechanisms of action [22]. In the same study, the authors reported increased levels of butyrate and propionate in the colon as a consequence of a higher amount of indigested carbohydrates reaching the distal gut.

Concerning the effect of tannins on the gut microbiota, very few data are available in the literature. Classical microbiological techniques have been used to investigate several groups of bacteria that are considered important for animal health [10]. We found only one study that considered tannins extracted from grapes, showing that the supplemented diet increased the abundance of *Lachnospiraceae*, *Clostridiales*, *Lactobacillus*, and *Ruminococcacceae* [23]. These groups of bacteria are recognised as major producers of short-chain fatty acids (SCFAs) in the human and animal guts. In particular, *Lachnospiraceae* and *Clostridiales* include the most important producers of butyric acid [24–26]. Butyrate plays an important role in the health of piglets during weaning, reducing intestinal inflammation and favouring the adaptation of the intestine to the change in diet [27]. Moreover, a reduction in butyrate-producing bacteria can promote the growth of pathogenic bacteria, such as *Salmonella* [28]. Thus, an increase in butyrate in the gut of weaning piglets could be an important factor in mediating the positive effects of tannins on animal health.

In a recent study, we evaluated tannins derived from chestnut and quebracho trees were evaluated for their capability to affect growth performance, blood metabolic profile, and faecal nitrogen concentration in post-weaned piglets [29]. Not surprisingly, we observed a higher fecal nitrogen excretion in tannin-fed piglets consistently with the known tannin property to form less digestible complexes with dietary proteins [30]. However, the performance of treated animals was comparable with that of controls, despite a very high dose of tannins administered. This result, in addition to the lower serum urea concentration detected in tannin-fed piglets, suggested a putative role of the gut microbiota in improving protein digestion and nitrogen utilization thereby supporting overall animal health. Therefore, in the present study we sought to highlight microbial changes occurring in the gut of weaned piglets that could mitigate the adverse effects of tannins on feed utilisation.

## Materials and methods

### Animals and sample collection

The present study was undertaken as part of an investigation of the effects of tannins on piglet gut microbiota composition and performance. A detailed description of the experimental animals, study design, and dietary treatments can be found in our previous study [29]. The trial was conducted in agreement with the Italian regulations on animal experimentation and ethics (DL 26/2014) and with the European regulation (Dir. 2010/6). Moreover, the trial was approved by the Animal Welfare Body of the University of Milan (number 31/2019). Briefly, a total of 120 weaned piglets (28 ± 2 days age; 50% female and 50% male), were allotted in randomized complete block design into two experimental groups: control group (CTR) and treatment group (TAN). There were 60 pigs per treatment with 6 replicate pens and 10 pigs per pen. The groups were homogeneous in terms of gender, weight and litter. After one week of adaptation (considered day 0, piglets were 35 days old), during which all animals received the same basal diet, the experimental diets were distributed *ad libitum* to all animals for 40 days. Experimental diets (Plurimix, Fabermatica, CR, Italy) were formulated according to animal requirements for the post-weaning phase (Ferraroni Mangimi SpA, Bonemerse, Italy). The two diets were isoenergetic and isoproteic and differed for the inclusion of 1.25% of tannin extract (contain 75 g of tannin/100 g of dry matter; Ch/Qu; Silvafeed Nutri P/ENC for Swine, Silvateam, Italy) from chestnut and quebracho trees in the treatment group. Both diets fulfilled the National Research Council (2012) [31] requirements for post-weaned piglets (S1 Table). Twelve piglets (2 piglets per pen) were randomly selected from each of the two dietary groups. During the entire experimental period, mortality was registered, and the incidence of diarrhea was calculated based on the number of piglets with clinical sign of diarrhea. Stools were individually collected from rectal ampulla at the end of the trial, which corresponded to day 40 of the dietary intervention (75-day-old piglets). Samples were immediately frozen in dry ice and then transferred to the laboratory for further analysis.

### Gas chromatographic analysis of faecal short-chain fatty acids

The extraction of short-chain fatty acids (SCFAs) was carried out starting with a sample of 3 g resuspended in 9 ml of distilled water by stirring. The suspension was centrifuged at 2000 rpm for 15 min, and then 2 ml of the obtained supernatant was added to 1 ml of a pivalic acid solution (internal standard, 1 g L^-1^ in distilled water) and 1 ml of a 0.12 M oxalic acid solution. After mixing, the suspension was centrifuged at 2000 rpm, and the resulting upper phase was microfiltered in vials.

Gas chromatographic analysis was carried out in a Shimadzu 2025 gas chromatograph equipped with an AOC-20i auto-sampler (Shimadzu Srl, Milan, Italy), FID detector, using a 30 m x 0.250 mm x 0.25 μm DB-FFAP capillary column (Agilent Technologies, Inc. Santa Clara, CA, USA). The temperatures of the injector and detector were 200 and 220 °C, respectively. The injection (1 μl of the sample) was carried out in split mode. The analysis was performed at a constant flow of carrier gas using the following cycle of temperature. The initial temperature of 60 °C was held for 5 min; the temperature was then raised to 160 °C at 5 °C min^-1^ and finally to 190 °C at 10 °C min^-1^, and this last temperature was held for 7 min.

Data acquisition and processing were performed using the LabSolutions Lite software (v5.82, Shimadzu Srl, Milan, Italy). SCFA identification was based on the retention time of an external standard, while pivalic acid was used as an internal standard.

### Bacterial DNA extraction, V3-V4 region amplification, and sequencing

Bacterial genomic DNA was extracted from 50 mg (wet weight) faecal samples using the FastDNA^™^ SPIN Kit for Soil (MP Biomedicals, Switzerland) following the manufacturer’s instructions. Quantification of the extracted DNA was carried out by a Qubit HS dsDNA fluorescence assay (Life Technologies, Carlsbad, CA, USA), and the quality check was performed using agarose gel electrophoresis. Amplification of the V3-V4 regions of the bacterial 16S rRNA gene was obtained using the primers 343F and 802R following the already described procedures [32]. The PCR products were checked by agarose gel electrophoresis and quantified using the Qubit HS dsDNA fluorescence assay (Life Technologies, Carlsbad, CA, USA) in order to prepare a pool of amplicons in which the PCR products of each sample were present in equimolar concentrations. The pool was then purified using a DNA Clean & Concentrator^™^-5 Kit (Zymo Research, Irvine, CA, USA).

Fasteris SA (Geneva, Switzerland) performed the sequencing of amplicons using Illumina’s MiSeq v3 platform in 2 x 300 bp mode.

Trimmomatic version 0.32 (http://www.usadellab.org/cms/index.php?page=trimmomatic) was used for quality filtering of raw reads (quality score ≥ 30) by sliding window trimming (window size: 4 base, quality: 15) and by dropping reads below a specified length (60 bases) [33]. Overlapped reads were mapped against the SILVA database (Version SSURef_NR99_115_tax_silva_DNA.fasta) using Burrows-Wheeler Alignment Tool version 0.7.5a (http://bio-bwa.sourceforge.net/). The package SAM tools was used to merge alignments and compute the number of reads mapped onto each OTU [34]. The data of the 16S rRNA gene sequences are available at the European Nucleotide Archive (ENA) (https://www.ebi.ac.uk) for tannin samples (accession numbers ERS5141801-ERS5141812) and control group samples (accession numbers ERS5141296-ERS5141307).

### Statistical analysis

Statistical analysis of the microbiota data was performed using the MicrobiomeAnalyst tool [35,36]. This tool allowed the calculation of alpha diversity based on the Chao 1, observed species, Simpson, and Shannon metrics. Significant differences between these indices were calculated using a t-test. Beta diversity across samples was calculated using the Bray–Curtis index and the PERMANOVA statistical methods. The EdgeR algorithm with an adjusted p-value cut-off, false discovery rate (FDR), of 0.05 was used to identify significant differences in taxa abundance of faecal bacteria between the two dietary groups. Concerning the prediction of the functional profiles of the bacterial communities across the two dietary groups, the operational taxonomic unit (OTU) table was uploaded in the MicrobiomeAnalyst software using the Marker Data Profiling option. The aim was to generate a Kyoto Encyclopedia of Genes and Genomes (KEGG) ontology (KO) assignment table using the Tax4Fun source in order to further analyse the data in the shotgun data profiling option. The differential relative abundance of genes across the two dietary groups was analysed using the EdgeR and Random Forest algorithms. Moreover, clusters of orthologous groups (COGs) of proteins were analysed using the same algorithms to identify the significant differences in COGs between the two dietary groups. Network analysis of faecal microbiota was performed using SparCC correlation coefficients as implemented in the R package SpiecEasi v1.1.0 [37] and visualised using qgraph v1.6.5 [38]. GraphPad Prism v8 (GraphPad Software, San Diego, CA, USA) was used to perform t-test and Spearman’s correlation analyses on the SCFA and serum urea data. For each genus, we examined the effect of the relative abundance on the recorded body weight. We fitted linear models separately for control and tannin-fed groups using body weight as response variable and the relative abundance of the focal genus as regressor. Further, the covariance between control and tannin-fed groups was evaluated including the interaction between relative abundance and group (control and tannin-fed animals) to the model. Analyses were carried out using R v4.0.2 [39].

## Results and discussion

### Microbiota composition and community diversity associated with tannin supplementation

**Fig 1:**
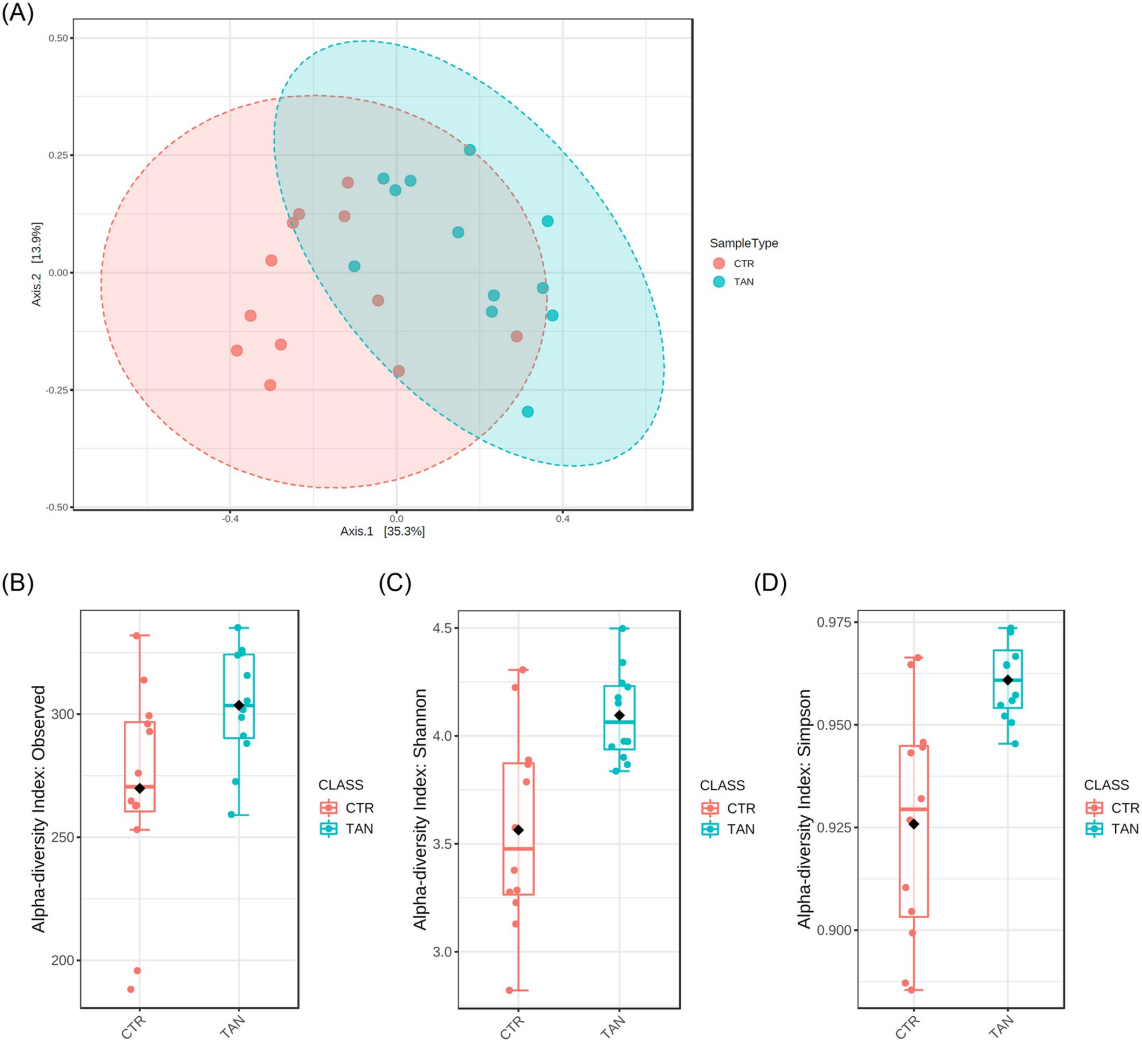
(A) Principal coordinate analysis (PCoA) plot based on Bray–Curtis distances of the intestinal microbial communities of weaning piglets fed on a diet with tannin (TAN) and the control group receiving a basal diet (CTR). (B) Observed species index (p = 0.03), (C) Shannon index (p = 0.02), and (D) Simpson index (p = 0.001). The index values are graphically presented by box plots, each of which represents the interquartile range, whereas the line inside the box represents the median.

A total of 2,694,143 reads were obtained after the filtering procedure, with an average sequence number for each sample of 112,255. As shown in the principal coordinate analysis (PCoA) ordination plot (Fig 1A), the tannin and control groups did not cluster separately, although PERMANOVA analysis indicated significant differences between the gut microbiota of piglets (R^2^ = 0.24, p < 0.001). To investigate the modification of the gut microbiota composition due to tannin supplementation, the alpha diversity of bacterial communities was calculated. The Chao 1 index did not differ between the control group (291.89 ± 41.8) and the tannin group (318.47 ± 23.0) (p = 0.06) (data not shown), whereas the observed species index was significantly higher in the tannin group (303.58 ± 23.1 vs. 269.83 ± 43.14, p = 0.03) (Fig 1B). The Shannon and Simpson indices, both reflecting the species richness and evenness, were significant higher in the tannin-supplemented animals (4.09 ± 0.21 vs. 3.56 ± 0.46, p = 0.02; 0.96 ± 0.01 vs. 0.93 ± 0.03, p = 0.001) (Fig 1C and 1D). In general, biologically diverse communities are thought to have high stability and resistance in the face of several disturbances and environmental changes [40].

**Fig 2:**
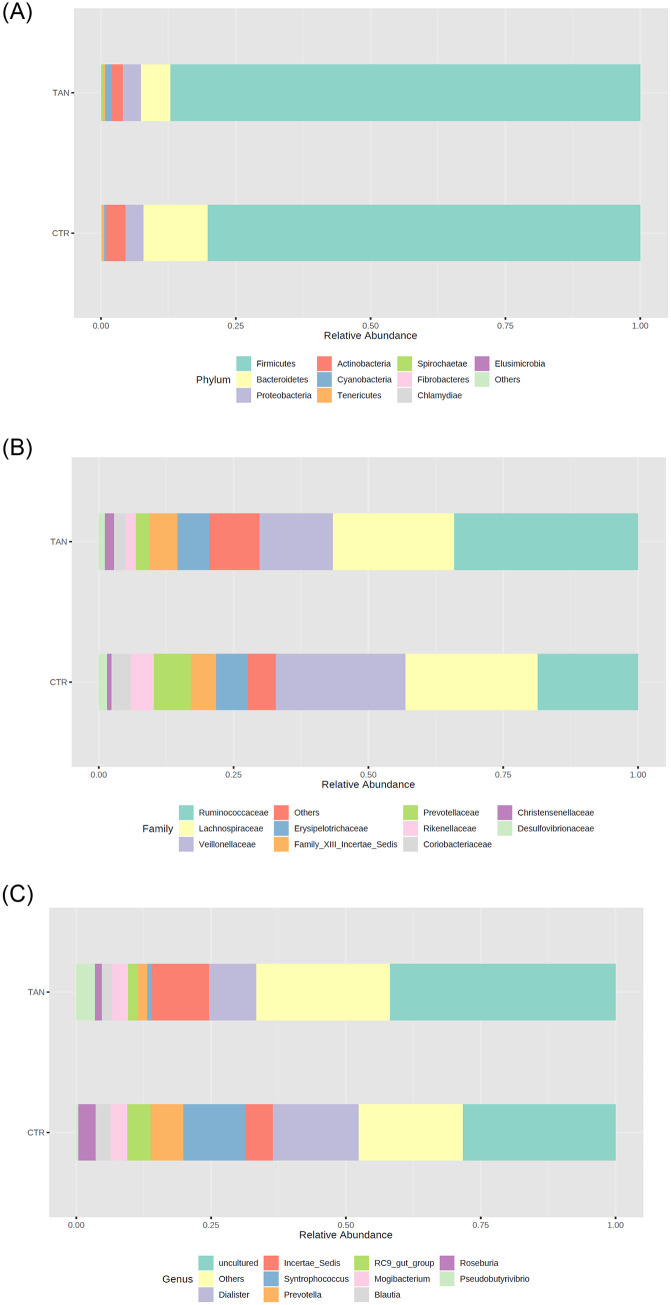
Relative abundances of phyla (A), families (B), and genera (C) observed in the tannin-supplemented group (TAN) compared with those of the control group (CTR). Only the top taxa are represented in the graphs.

**Fig 3:**
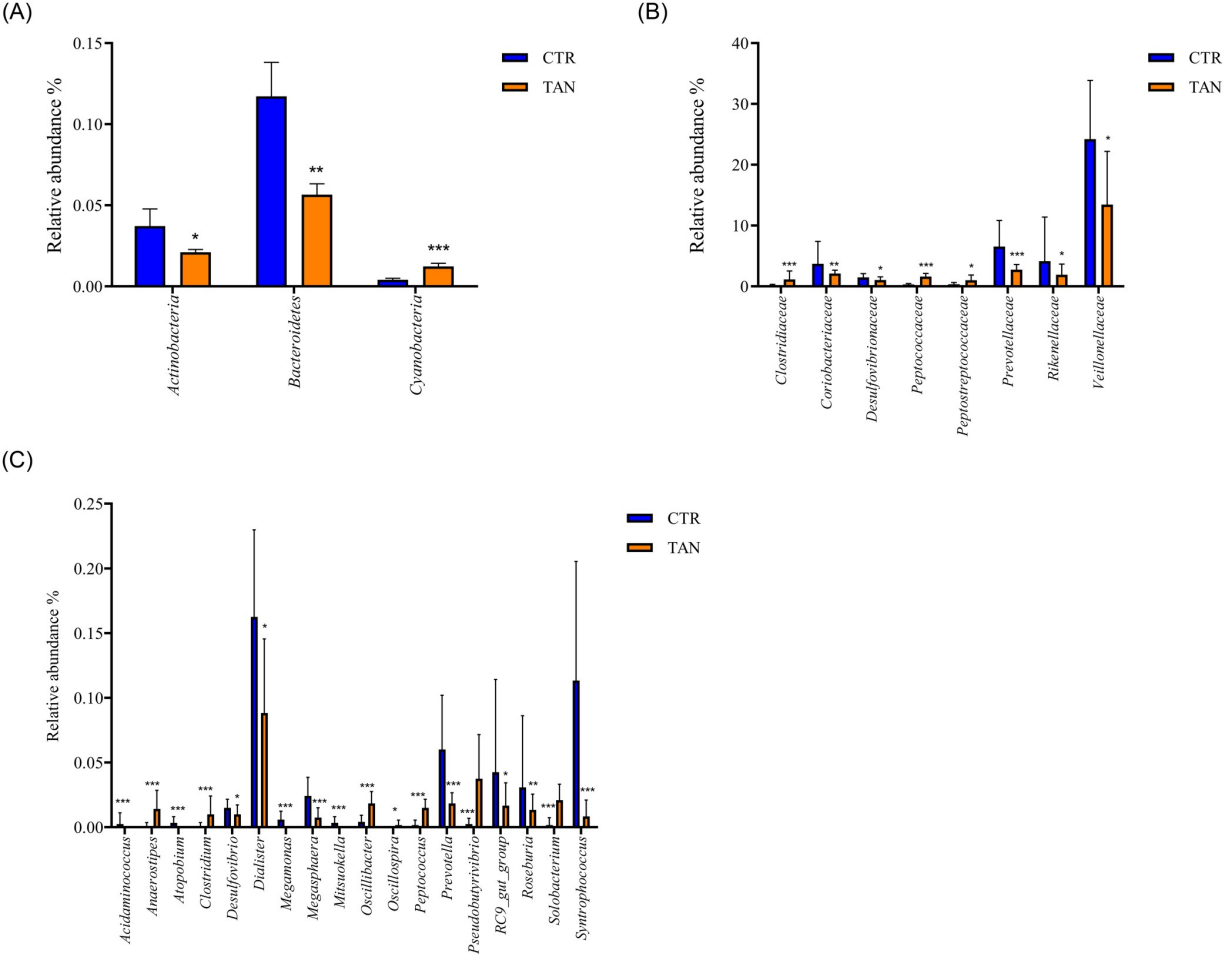
Relative abundance of fecal bacterial populations at phylum (A), family (B) and genus (C) levels in tannin-fed (TAN) and control (CTR) piglets. Only taxa with a mean relative abundance higher than 0.002% are shown. p value significance levels were reported as * p ≤ 0.05; ** p ≤ 0.01; *** p ≤ 0.001.

Regarding the gut microbiota composition at the phylum level (Fig 2A), a significant increase in *Cyanobacteria* and *Spirochaetae* (FDR < 0.001 and FDR = 0.012, respectively) was detected in stools collected from the tannin-supplemented animals, whereas *Bacteroidetes* and *Actinobacteria* (FDR = 0.002 and FDR = 0.02, respectively) were reduced (Fig 3A and S2 Table). So far, no data on piglet gut microbiota modulation associated with tannins are available in the literature; a reduction in the faecal levels of *Bacterioidetes* was detected in chickens fed on a diet enriched with chestnut tannins [41]. Moreover, at the family level, several differences were found between the two dietary groups of animals, as shown in Fig 2B. Tannin supplementation strongly increased the relative abundance of *Peptococcaceae* and *Clostridiaceae*, whereas a reduction was observed in the relative abundance of *Prevotellaceae* and *Eubacteriaceae*. The microbiota of tannin-fed piglets had fewer sequences from *Coriobacteriaceae*, *Desulfovibrionaceae*, *Veillonellaceae*, *Rikenellaceae*, and *Deferribacteraceae*, whereas it was enriched in *Spirochaetaceae* and *Peptostreptococcaceae* (Fig 3B and S2 Table).

At the genus level, many significant differences (FDR<0.05) were found between the two dietary groups (Fig 2C). Therefore, only the most significant ones were taken into consideration, namely those showing an FDR <0.0001. In the tannin-supplemented animals, the genera *Shuttleworthia*, *Pseudobutyrivibrio*, *Peptococcus*, *Anaerostipes*, and *Solobacterium* were significantly increased. Conversely, the genera *Syntrophococcus*, *Atopobium*, *Mitsuokella*, *Sharpea*, and *Prevotella* were significantly decreased in samples collected from the tannin-supplemented group of animals (Fig 3C and S3 Table).

**Fig 4:**
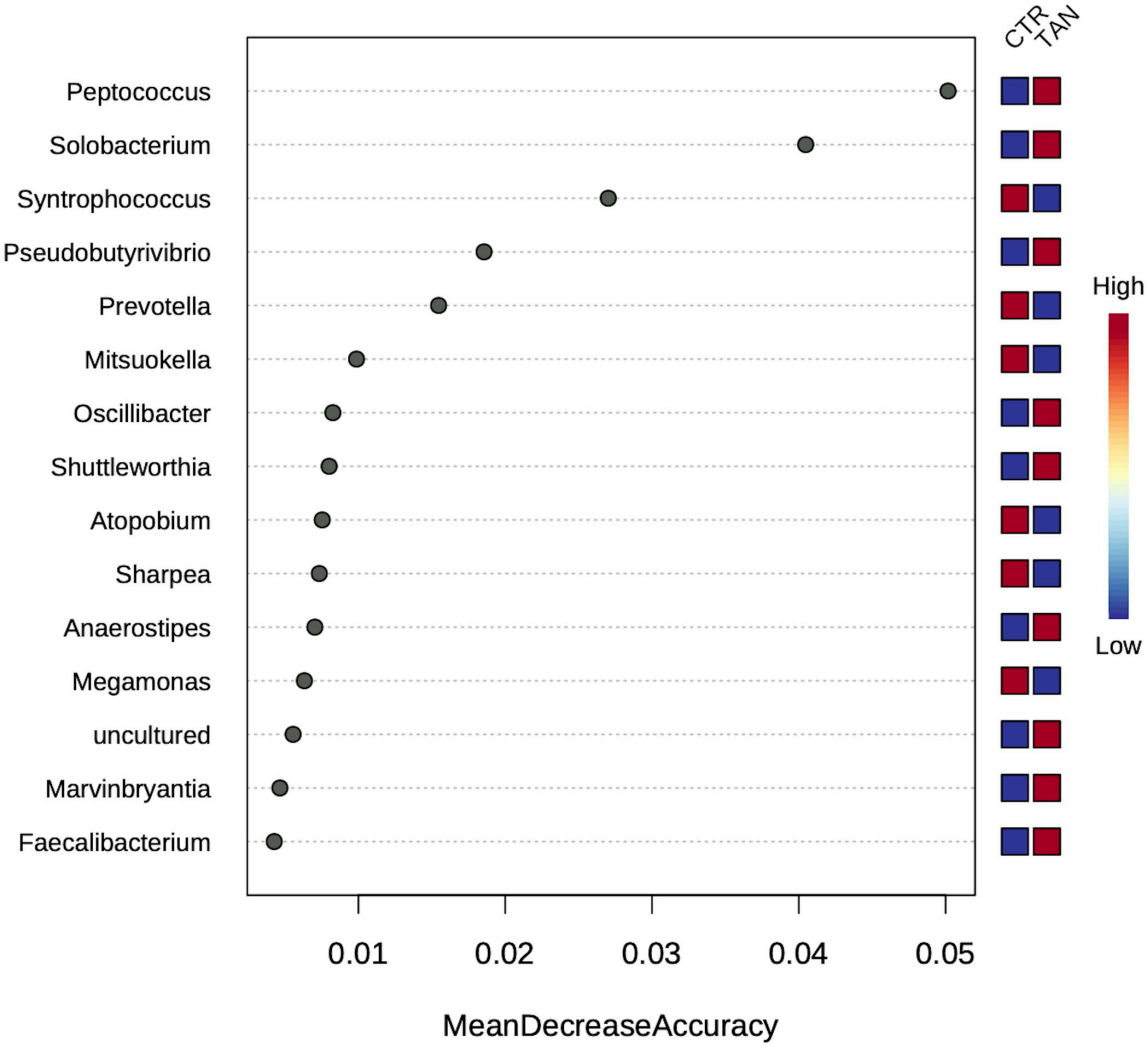
The most discriminant genera between the control- and tannin-sample sequences as sorted by Random Forest analysis using the mean decrease in accuracy.

Given the high number of statistically significant different taxa between the two dietary groups, a random forest analysis was performed to identify the bacterial populations that were important in differentiating the tannin-fed versus control animals. Random forest analysis revealed that *Peptococcaceae*, an unidentified member of the human gut metagenome, and *Prevotellaceae* were the most discriminant taxa at the family level, while *Peptococcus*, *Solobacterium*, *Syntrophococcus*, *Pseudobutyrivibrio*, and *Prevotella* had the highest importance scores at the genus level (Fig 4). Previous studies reported that *Peptococcus* was more abundant in pigs showing high performance, suggesting a positive correlation with body weight [42–44]. On the other hand, in piglets, a higher risk of post-weaning diarrhoea has been associated with an increase in the intestinal levels of *Prevotella* concurrently with a decrease in non-pathogenic *Escherichia coli* and beneficial *Firmicutes* [45,46]. According to our results, tannins could contribute to preventing an increase in *Prevotella* in the gut. In our experimental animals, we observed transient signs of diarrhoea after seven days of treatment; the highest incidence was detected at 14–28 days with slightly lower percentages in tannin-fed piglets vs controls, though such differences were not significant [29]. Other populations that were modulated by tannin included genera such as *Butyrivibrio*, *Pseudobutyrivibrio*, *Oscillospira*, and *Oscillibacter*. The genus *Butyrivibrio* has been detected only in animals showing high performance, suggesting that the high efficiency of this bacterial genus in degrading complex carbohydrates could positively impact animal feed conversion [47]. Regarding *Oscillibacter*, this genus was correlated with a higher weight gain [47]. The genus *Oscillospira*, on the other hand, was positively associated with harder stools [48], whereas its decrease in human gut microbiota was correlated with an inflammatory status [49]. Both properties could have positive implications during the weaning period, so the potential impact of these bacterial populations on pig gut function and physiology warrants further investigation. Moreover, *Solobacterium* was increased in the tannin group, although it has been described as being associated with low feed intake in piglets [47]. Nevertheless, increased levels of this genus, together with other anaerobic bacteria, were correlated with positive effects in new-born piglets treated with antibiotics [50]. To measure the relationship between bacterial relative abundance and animal body weight for both control and tannin-fed groups, we fitted linear models to observed data. *Mucispirillum* was the only genus for which a significant interaction between the relative proportion and the experimental group was recorded (p = 0.01). The interaction effect in *Butyrivibrio* and *Oscillospira* tended to significance (p ~ 0.07). Notably, *Mucispirillum* showed significant association (R = 0.88, p = 0.00017) only within the tannin-fed group indicating that its reduction was related to a lower animal body weight. *Mucispirillum* is an inhabitant of the intestine of rodents and other animals, including pigs. It is considered an immunogenic commensal bacterium able to adhere to the intestinal mucus and to interact with the host immune system [51].

We used the Spearman’s rank correlation coefficient to measure the association between plasma urea, nitrogen excretion and fecal bacterial genera. Correlation analysis revealed positive correlations between *Oscillospira* (r = 0.74, p = 0.00004), *Faecalibacterium* (r = 0.64, p = 0.0008), *Peptococcus* (r = 0.69, p = 0.0002), *Anaerostipes* (r = 0.61, p = 0.0016), *Solobacterium* (r = 0.53, p = 0.007) and nitrogen excretion. This parameter showed negative correlations with *Syntrophococcus* (r = -0.59, p = 0.002), *Prevotella* (r = -0.54, p = 0.007), *Mitsuokella* (r = -0.51, p = 0.01). The fundamental role of intestinal nitrogen in driving the interaction between the host and the gut microbiome has been already established [52]. Based on a predictive model analysis, nitrogen availability was reported to exert a primary control in the assembly of gut microbial communities by limiting microbial competition for carbon sources [52]. In addition, a negative correlation was found between urea and *Anaerostipes* (r = -0.779; p<0.0001), *Pseudobutyrivibrio* (r = -0.687; p = 0.0002), *Faecalibacterium* (r = -0.500; p = 0.013), *Oscillibacter* (r = -0.436; p = 0.033), *Oscillospira* (r = -0.522; p = 0.009), *Shuttleworthia* (r = -0.484; p = 0.017) and *Solobacterium* (r = -0.543; p = 0.006). All these taxa increased in tannin-fed piglets as compared with controls. Conversely, positive correlations were detected between this blood parameter and genera that were less abundant in tannin-fed animals, i.e. *Atopobium* (0.685; p = 0.0002), *Syntrophococcus* (r = 0.580; p = 0.003), *Acidaminococcus* (r = 0.471; p = 0.02), *Howardella* (r = 0.545; p = 0.006), *Megamonas* (r = 0.410; p = 0.046) and *Megasphaera* (r = 0.520; p = 0.009). Remarkably, a number of studies have reported that lower serum urea concentration is linked to a more efficient utilization of protein/nitrogen by animals [53,54]. Although correlation does not imply causation, the association between this marker and fecal bacterial taxa found in this the present study suggests a potential involvement of tannin-driven microbial population shifts in whole-animal nitrogen balance in piglets.

### Short chain fatty acids content in stools

**Fig 5:**
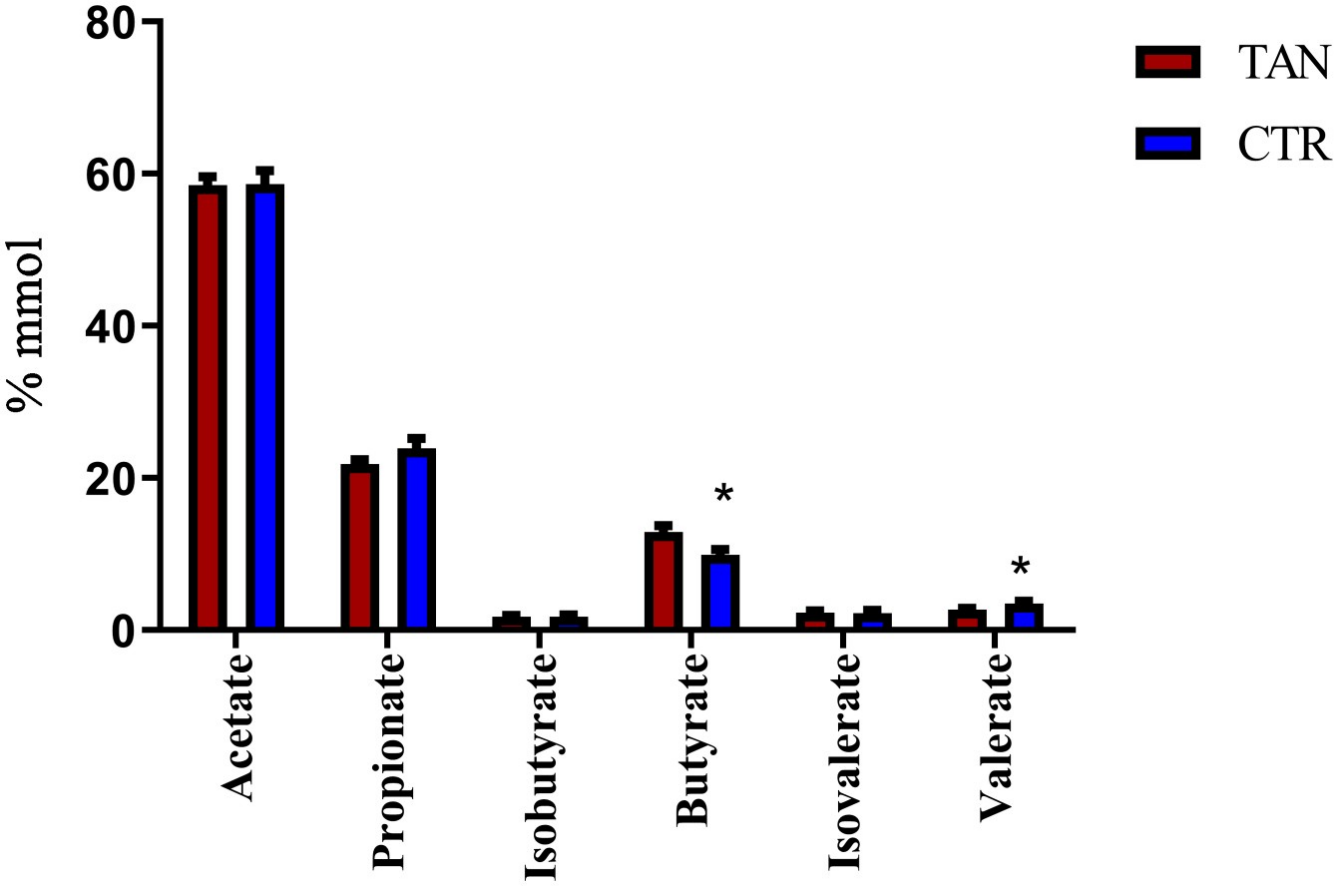
Proportion of short-chain fatty acids in control (CTR) and tannin-fed (TAN) piglets. The * indicates a p value ≤0.05.

The determination of SCFAs in piglet stools revealed a significant increase in butyrate proportion in the tannin group (12.88 ± 3.11) as compared with the control group (9.93 ± 2.41, p = 0.018). In addition, a significant decrease in valerate (2.69 ± 0.62 vs. 3.51 ± 1.09, p = 0.044) was also detected in the tannin-fed animals (Fig 5). The total concentration of SCFA was similar (p = 0.1413) in both groups of experimental piglets (CTR 0.0723 mmol/g±0.014; TAN 0.082 mmol/g±0.017).

The importance of butyrate for the host’s gut health is well established, and its positive role in the intestinal mucosa and immune responses has been described in weaning piglets [27]. Results of the Spearman’s correlation analysis indicated that there was a significant positive association between butyrate and *Pseudobutyrivibrio* (r = 0.669, p = 0.0003), *Atopobium* (r = -0.527, p = 0.008), and *Anaerostipes* (r = 0.509, p = 0.011). As concerns valerate, it can be produced by the degradation of lactate, as shown for some strains of *Megasphaera elsdenii* in pigs [55], but small amounts of this chain fatty acid can be formed by protein and amino acid degradation by other intestinal bacteria [53]. Based on our correlation results, fecal valerate content displayed a weak positive association with the levels of *Megasphaera* (r = 0.44, p = 0.031) and *Desulfovibrio* (r = 0.44 p = 0.034). *Megasphaera* showed the same positive correlation in the proximal colon of pigs fed a trans-glycosylated starch diet [56]. Overall, valerate has been poorly investigated, but it has been described as an alternative source of energy for colonocytes in pigs [54].

### Co-occurrence networks of the intestinal microbiota of control and tannin group animals

**Fig 6:**
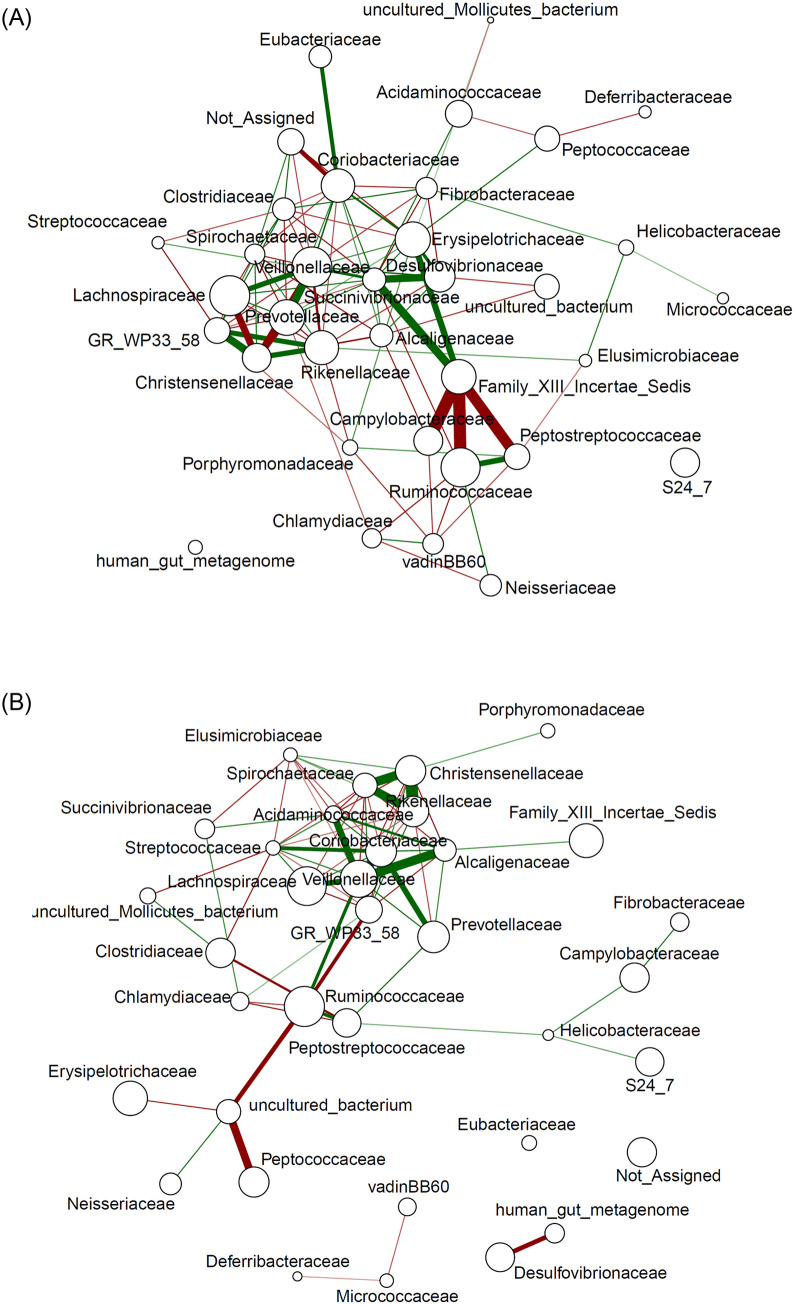
SparCC co-occurrence network analysis at the family level of the control (A) and the tannin (B) group microbiota. The node size represents the relative abundance of families. Lines represent correlations between the nodes they connect. The green colour of an edge indicates positive correlations, whereas the red colour denotes negative correlations. The colour intensity and line thickness indicate the correlation magnitude. Only significant correlations (p < 0.05) are shown.

To explore the dynamics of gut bacterial interactions across diets, a bacterial community network analysis was performed for each group of animals (Fig 6A and 6B). Only significant correlations (p < 0.05) are shown.

In the network of the control group, *Desulfovibrionaceae*, *Veillonellaceae*, *Lachnospiraceae*, *Prevotellaceae*, *Succinivibrionaceae*, *Erysipelotrichaceae*, and Family XIII Incertae Sedis were positively correlated with each other. The strongest correlations were found between *Veillonellaceae* and *Prevotellaceae* (SparCC = 0.86), between *Succinivibrionaceae* and *Desulfovibrionaceae* (SparCC = 0.81), and between *Succinivibrionaceae* and Family XIII Incertae Sedis (SparCC = 0.78). Conversely, *Christensenellaceae* showed strong negative correlations with *Lachnospiraceae* (SparCC = −0.89) and *Prevotellaceae* (SparCC = −0.94). At the edge of the network, Family XIII Incertae Sedis was negatively correlated with *Peptostreptococcaceae* (SparCC = −0.74), *Ruminococcaceae* (SparCC = −0.96), and *Campylobacteriaceae* (SparCC = −0.95).

In the tannin group samples, the families *Veillonellaceae*, *Lachnospiraceae*, and *Coriobacteriaceae* occupy the central part of the network, as for the previous group. Moreover, the *Spirochaetaceae* family, which characterised the network of the tannin group, showed strong positive correlations with *Christensenellaceae* (SparCC = 0.88) and *Rikenellaceae* (SparCC = 0.87). The presence of *Acidaminococcaceae* and *Alcaligenaceae* characterised the co-occurrence network of the tannin group. *Veillonellaceae* showed strong positive correlations with both *Acidaminococcaceae* (SparCC = 0.85) and *Alcaligenaceae* (SparCC = 0.80).

Finally, Family XIII Incertae Sedis was not present in the central part of the tannin group network and did not display any significant correlations with other families.

These findings confirmed that tannin supplementation influenced both the composition of the gut microbiota of piglets and the interactions between different bacterial groups, mainly hydrogen-consuming populations such as *Desulfovibrio*. This decrease could be due to a particular sensitivity of this genus to tannins and/or its derivative compounds, as well as to a different hydrogen balance in the gut environment.

### Functional prediction

**Fig 7:**
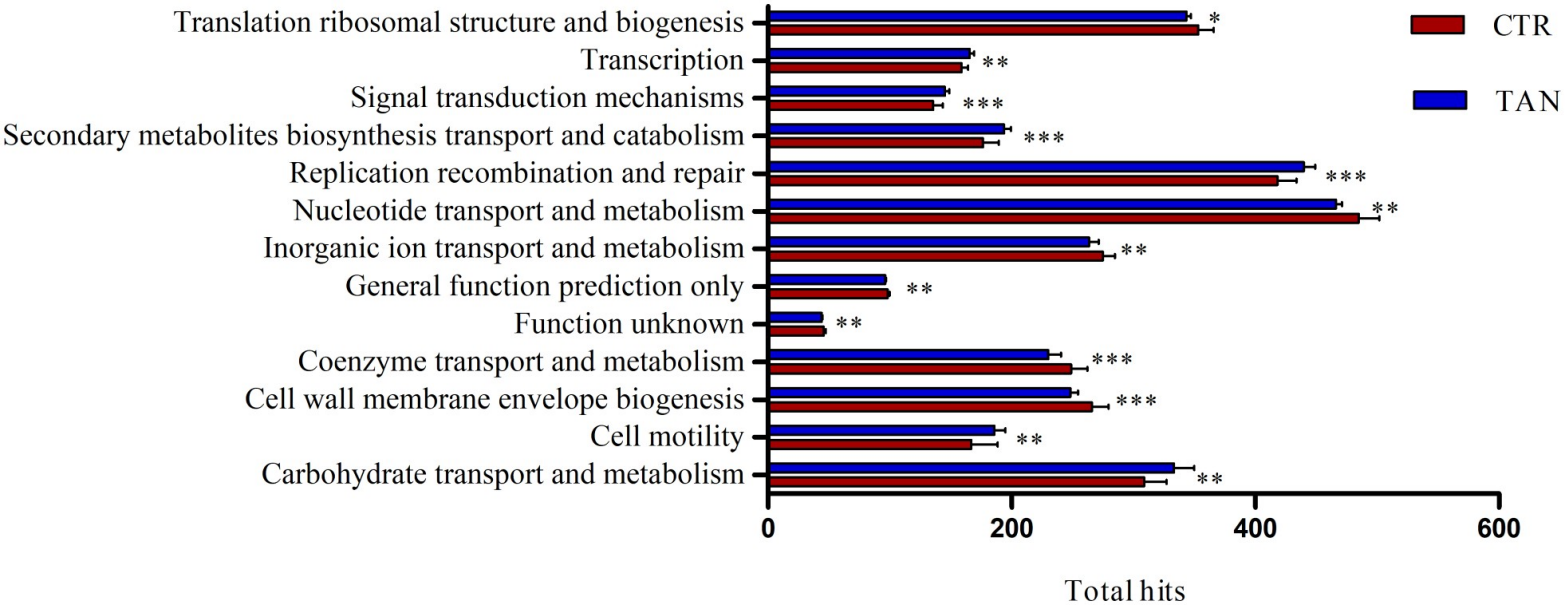
Significant different orthologous groups of proteins between the tannin (TAN) and control (CTR) piglets. p value significance levels were reported as * p ≤ 0.05; ** p ≤ 0.01; *** p ≤ 0.001.

The COG functional category profiles were analysed to identify significant differences between the two groups of samples (Fig 7 and S4 Table). More specifically, a significant reduction in genes involved in lipopolysaccharide (LPS) biosynthesis (i.e. K00912, K00677, K02527, K00748, and K03272) was found in the tannin group. LPSs are components of the outer membrane of Gram-negative bacteria that are often associated with inflammation. They are able to induce inflammation by activating the host’s immune cells [57], although LPSs seem to play a role in facilitating the host’s tolerance of gut microbiota [58]. The reduction in LPS genes in our samples could be due to a decrease in Gram-negative bacteria numbers, such as *Bacteroidetes* and particularly *Prevotella*, following the supplementation of tannins. These results could suggest a reduction in components capable of inducing immune responses, and, consequently, of affecting the inflammation status in piglets fed with tannins. Further analysis is required in order to confirm this hypothesis.

Moreover, in the tannin group samples, the genes encoding for enzymes associated with propanoate (i.e. K00169, K00170, K00172, and K01026) and butanoate (K00248, K14534, K00169, K00170, K04072, K00172, and K00171) metabolism were enriched. In particular, the enriched genes of the butanoate pathway were genes encoding for enzymes involved in carbohydrate fermentation. Conversely, genes involved in the butyrate conversion from succinate were less represented in the tannin group. For instance, one genus that was decreased in the tannin-supplemented group was *Prevotella*, a highly efficient fibre degrader producing succinate. Moreover, in the same group of samples, a reduction in genes encoding for phosphotransbutyrylase and butyrate kinase was detected, while genes encoding for butyryl-CoA:acetate-CoA transferase were enriched. Three of the genes that were enriched in the tannin samples encoded for common oxidoreductases in both the butanoate and the propanoate pathway. Overall, the outcome of the in silico analysis indicates a different balance of metabolic activities in the gut microbiota of animals fed on tannins, as a result of the modulation of bacterial populations that produce butyrate via different metabolic routes. Indeed, *Acidaminococcus*, *Megasphaera*, *Pseudoramibacter*, and *Roseburia* showed a statistically significant reduction, whereas *Shuttleworthia*, *Anaerostipes*, *Faecalibacterium*, *Pseudobutyrivibrio*, *Butyrivibrio*, *Oscillibacter*, and *Oscillospira* were increased in the tannin group [25,49,59,60]. Therefore, the species-specific functionalities and the intra-species diversity can explain the modulation of butyrate metabolism. Notably, the dietary regulation of the metabolic pathways underlying butyrate production in tannin-fed piglets seems to result in an overall positive balance, as indicated by the higher production yield of butyric acid in the tannin group as described above.

Moreover, our analysis revealed a significant increase in carbohydrate metabolism that has been associated with low feed efficient pigs [47,61].

### Conclusion

As far as we know, this is the first investigation of the effects of quebracho and chestnut tannins on the gut microbiota of weaned piglets. In this study, tannin supplementation caused changes in the composition of the gut microbiota and specifically modulated the populations of butyrate-producing bacteria. The major limitation of this work is that the animals involved in the trial did not show any clear health problems, so we could not evaluate the tannins’ potential capability to prevent diseases associated with weaning. The next step could therefore be testing the tannin mixture in artificially infected piglets.

## Supporting information

S1 Table Ingredients and chemical composition of experimental diets. (DOCX)

S2 Table Differentially abundant families between the control and tannin groups of piglets. Abbreviations: FDR = false discovery rate <0.05; FC = Fold change. Positive log2 fold change is the relative abundance in the tannin group compared with the control group.
(DOCX)

S3 Table Differentially abundant genera between the tannin and control groups of animals. Abbreviations: FDR = false discovery rate <0.05. FC = Fold change. Positive log2 fold change is the relative abundance in the tannin group compared with the control group.
(DOCX)

S4 Table Significant different orthologous groups of proteins between the tannin and control groups. Abbreviations: FDR = false discovery rate <0.05. FC = Fold change. Positive log2 fold change is the relative abundance in the tannin group compared with the control group.
(DOCX)
