# Supplementary material for: A mixture of quebracho and chestnut tannins drives butyrate-producing bacteria populations shift in the gut microbiota of weaned piglets
Source: PLoS One. 2021 Apr 29;16(4):e0250874. doi: 10.1371/journal.pone.0250874 (PMC8084250; doi:10.1371/journal.pone.0250874)
Supplement: S1 Table — (DOCX) [file pone.0250874.s001.docx]

**S1 Table** Ingredients and chemical composition of experimental diets

|  | Diet^1^ | |
| --- | --- | --- |
| Ingredients (g/kg as fed basis) | **CTR** | **TAN** |
| Barley, meal | 251.50 | 241.80 |
| Wheat, meal | 194.10 | 192.20 |
| Corn, flakes | 140.30 | 135.00 |
| Corn, meal | 48.50 | 48.50 |
| Soybean, meal | 46.50 | 47.50 |
| Soy protein concentrates | 41.10 | 41.00 |
| Biscuits, meal | 40.00 | 40.00 |
| Dextrose monohydrate | 35.00 | 35.00 |
| Wheat middlings | 43.20 | 44.20 |
| Whey protein concentrate | 30.00 | 30.00 |
| Fish, meal | 25.00 | 25.00 |
| Milk whey, powder | 25.00 | 25.00 |
| Coconut oil | 10.00 | 10.00 |
| Soybean oil | 10.00 | 12.50 |
| Plasma, meal | 10.00 | 10.00 |
| Organic acids^2^ | 10.00 | 10.00 |
| Di-calcium phosphate | 8.50 | 8.50 |
| Animal fats | 7.00 | 7.00 |
| L-Lysine | 5.00 | 5.00 |
| Benzoic acid | 4.00 | 4.00 |
| L-Threonine | 3.50 | 3.50 |
| DL-Methionine | 3.50 | 3.50 |
| Sodium chloride | 2.70 | 2.70 |
| Vitamins^3^ | 2.50 | 2.50 |
| L-Valine (96.5%) | 1.50 | 1.50 |
| L-Tryptophan | 0.80 | 0.80 |
| Flavouring^4^ | 0.40 | 0.40 |
| Copper sulphate | 0.40 | 0.40 |
| Tannin extract^5^ | - | 12.50 |
| Calculated nutrient levels^6^ (% as fed basis) |  |  |
| Crude protein | 16.92 | 16.88 |
| Ether extract | 5.06 | 5.19 |
| Crude fibre | 3.15 | 3.22 |
| Ashes | 5.10 | 5.10 |
| Digestible energy^7^ (Mcal/Kg) | 3.43 | 3.43 |

^1^ CTR: basal diet, TAN: basal diet supplemented with tannin extract (1.25%).

^2^ Organic Acids: formic acid, sodium formate, sorbic acid, orthophosphoric acid, calcium formate, citric acid, and fumaric acid.

^3^ Vitamins and vitamin-like compounds per kg: 10,000 UI vitamin A, 1000 UI vitamin D3, 100 mg UI vitamin E, 3 mg vitamin B1, 96.3 mg vitamin B2, 5.8 mg vitamin B6, 27 mg vitamin B5, 0.040 mg vitamin B12, 4.8 mg vitamin K3, 0.19 mg biotin, 35 mg niacinamide, 1.4 mg folic acid, 120 mg choline chloride, 70 mg betaine chloride, 108 mg Fe as FeCO3, 38.5 mg Mn as MnO2, 112 mg Zn as ZnO, 19.3 Cu as CuSO4, 0.58 I as Ca(IO3)2, 0.29 Se as Na2SeO3.

^4^ Vanilla flavouring.

^5^ Commercial chestnut and quebracho tannin extract (Silvafeed^®^ Nutri P, Silvateam, Italy).

^6^Nutrients content were calculated using Plurimix software (Fabermatica, CR, Italy).

^7^Digestible energy estimated following NRC (2012).
