# Supplementary material for: A mixture of quebracho and chestnut tannins drives butyrate-producing bacteria populations shift in the gut microbiota of weaned piglets
Source: PLoS One. 2021 Apr 29;16(4):e0250874. doi: 10.1371/journal.pone.0250874 (PMC8084250; doi:10.1371/journal.pone.0250874)
Supplement: S2 Table — Abbreviations: FDR = false discovery rate <0.05; FC = Fold change. Positive log2 fold change is the relative abundance in the tannin group compared with the control group. (DOCX) [file pone.0250874.s002.docx]

**S2 Table**. Differentially abundant families between the control and tannin groups of piglets. Abbreviations: FDR= false discovery rate <0.05; FC= Fold change. Positive log2 fold change is the relative abundance in the tannin group compared with the control group.

| Phylum | log2 FC | FDR |
| --- | --- | --- |
| *Cyanobacteria* | 1.8569 | 0.000225 |
| *Bacteroidetes* | -1.0093 | 0.002102 |
| *Spirochaetae* | 2.256 | 0.012288 |
| *Actinobacteria* | -1.1926 | 0.023065 |
| *Deferribacteres* | -0.97977 | 0.060334 |
| Family | **log2 FC** | **FDR** |
| *Peptococcaceae* | 2.2054 | 8.13E-09 |
| *Prevotellaceae* | -1.7474 | 9.37E-05 |
| *Eubacteriaceae* | -5.0904 | 0.000504 |
| *Clostridiaceae* | 2.8157 | 0.000942 |
| *Acidaminococcaceae* | -3.4958 | 0.000942 |
| uncultured_*Mollicutes*_bacterium | 2.3777 | 0.001199 |
| *Coriobacteriaceae* | -1.581 | 0.002862 |
| Not_Assigned | 1.4825 | 0.002862 |
| human_gut_metagenome | 2.4695 | 0.011972 |
| *Desulfovibrionaceae* | -0.97618 | 0.017875 |
| *Veillonellaceae* | -1.2691 | 0.019263 |
| *Rikenellaceae* | -1.3343 | 0.026373 |
| *Deferribacteraceae* | -1.2286 | 0.030414 |
| *Spirochaetaceae* | 1.9212 | 0.034031 |
| *Peptostreptococcaceae* | 1.3571 | 0.047621 |
