# Supplementary material for: A mixture of quebracho and chestnut tannins drives butyrate-producing bacteria populations shift in the gut microbiota of weaned piglets
Source: PLoS One. 2021 Apr 29;16(4):e0250874. doi: 10.1371/journal.pone.0250874 (PMC8084250; doi:10.1371/journal.pone.0250874)
Supplement: S3 Table — Abbreviations: FDR = false discovery rate <0.05. FC = Fold change. Positive log2 fold change is the relative abundance in the tannin group compared with the control group. (DOCX) [file pone.0250874.s003.docx]

**S3 Table**. Differentially abundant genera between the tannin and control groups of animals. Abbreviations: FDR= false discovery rate <0.05. FC= Fold change. Positive log2 fold change is the relative abundance in the tannin group compared with the control group.

| Genus | log2 FC | FDR |
| --- | --- | --- |
| *Shuttleworthia* | 4.4402 | 4.18E-06 |
| *Syntrophococcus* | -4.7316 | 4.18E-06 |
| *Atopobium* | -4.0962 | 6.30E-06 |
| *Pseudobutyrivibrio* | 2.8196 | 6.30E-06 |
| *Peptococcus* | 2.0023 | 7.57E-06 |
| *Mitsuokella* | -3.2245 | 1.05E-05 |
| *Sharpea* | -4.8289 | 1.05E-05 |
| *Prevotella* | -2.2012 | 1.23E-05 |
| *Anaerostipes* | 3.0896 | 7.15E-05 |
| *Solobacterium* | 2.7762 | 7.54E-05 |
| *Acidaminococcus* | -3.8175 | 0.000115 |
| *Megamonas* | -3.7222 | 0.000122 |
| *Megasphaera* | -2.1381 | 0.000186 |
| *Pseudoramibacter* | -4.7715 | 0.000407 |
| *Faecalibacterium* | 1.8584 | 0.000681 |
| *Clostridium* | 2.776 | 0.000695 |
| *Oscillibacter* | 1.7001 | 0.000978 |
| *Butyrivibrio* | 3.199 | 0.002217 |
| *Dialister* | -1.513 | 0.006128 |
| *Roseburia* | -1.9688 | 0.009045 |
| *Oscillospira* | 1.481 | 0.013524 |
| *Howardella* | -1.3444 | 0.020488 |
| *Desulfovibrio* | -1.0091 | 0.032081 |
| *RC9_gut_group* | -1.4789 | 0.038221 |
| *Mucispirillum* | -1.1901 | 0.038912 |
