# Supplementary material for: A mixture of quebracho and chestnut tannins drives butyrate-producing bacteria populations shift in the gut microbiota of weaned piglets
Source: PLoS One. 2021 Apr 29;16(4):e0250874. doi: 10.1371/journal.pone.0250874 (PMC8084250; doi:10.1371/journal.pone.0250874)
Supplement: S4 Table — Abbreviations: FDR = false discovery rate <0.05. FC = Fold change. Positive log2 fold change is the relative abundance in the tannin group compared with the control group. (DOCX) [file pone.0250874.s004.docx]

**S4 Table**. Significant different orthologous groups of proteins between the tannin and control groups. Abbreviations: FDR= false discovery rate <0.05. FC= Fold change. Positive log2 fold change is the relative abundance in the tannin group compared with the control group.

| Orthologous Group | log2 FC | FDR |
| --- | --- | --- |
| Cell wall membrane envelope biogenesis | -0.10281 | 0.000269 |
| Coenzyme transport and metabolism | -0.11612 | 0.000269 |
| Signal transduction mechanisms | 0.093233 | 0.00038 |
| Secondary metabolites biosynthesis transport and catabolism | 0.13138 | 0.000876 |
| Replication recombination and repair | 0.067267 | 0.000989 |
| Carbohydrate transport and metabolism | 0.10382 | 0.001196 |
| Inorganic ion transport and metabolism | -0.06637 | 0.002538 |
| Nucleotide transport and metabolism | -0.06132 | 0.002673 |
| Transcription | 0.054591 | 0.004896 |
| General function prediction only | -0.03855 | 0.004896 |
| Cell motility | 0.15219 | 0.006391 |
| Function unknown | -0.05379 | 0.007714 |
| Translation ribosomal structure and biogenesis | -0.04413 | 0.01669 |
